# Supplementary material for: Induced dipole moments in amorphous ZnCdS catalysts facilitate photocatalytic H2 evolution
Source: Nat Commun. 2024 Mar 23;15:2600. doi: 10.1038/s41467-024-47022-z (PMC10960824; doi:10.1038/s41467-024-47022-z)
Supplement: Supplementary file 1 — Supplementary Information [file 41467_2024_47022_MOESM1_ESM.pdf]

Supplementary Information

Induced dipole moments in amorphous ZnCdS catalysts facilitate photocatalytic H_2 evolution

Xin Wang[1], Boyan Liu[1], Siqing Ma[1], Yingjuan Zhang[1], Lianzhou Wang[2,*], Gangqiang Zhu[3,*], Wei Huang[1,*] & Songcan Wang[1,*]

[1]Frontiers Science Center for Flexible Electronics, Xi'an Institute of Flexible Electronics (IFE), Northwestern Polytechnical University, 127 West Youyi Road, Xi'an 710072, China

[2] Nanomaterials Centre, Australian Institute for Bioengineering and Nanotechnology and School of Chemical Engineering, The University of Queensland, Brisbane, Queensland 4072, Australia.

[3] School of Physics and Information Technology, Shaanxi Normal University, Xi'an 710062, China.

* Corresponding authors E-mail: l.wang@uq.edu.au (L. Wang), zgq2006@snnu.edu.cn (G. Zhu), iamwhuang@nwpu.edu.cn (W. Huang), iamscwang@nwpu.edu.cn (S. Wang)

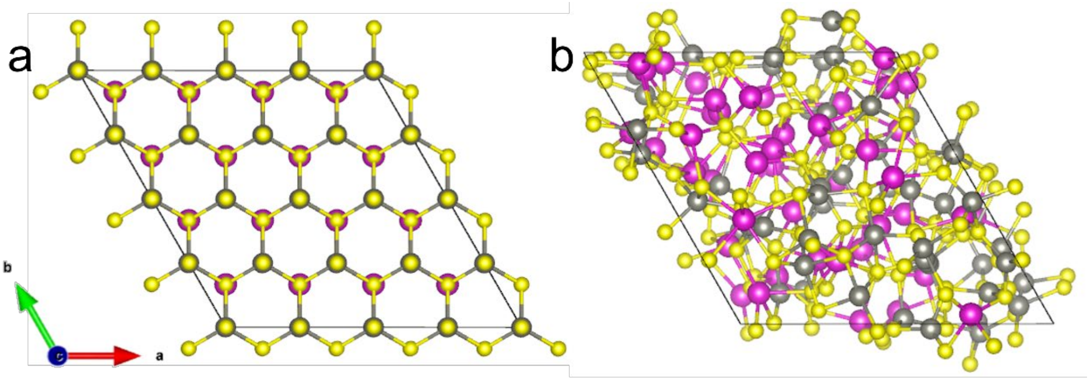

Fig. S1. The top view of the atom arrangement of different samples. a CZCS and **b** AZCS (Zn: grey spheres, Cd: pink spheres, S: yellow spheres).

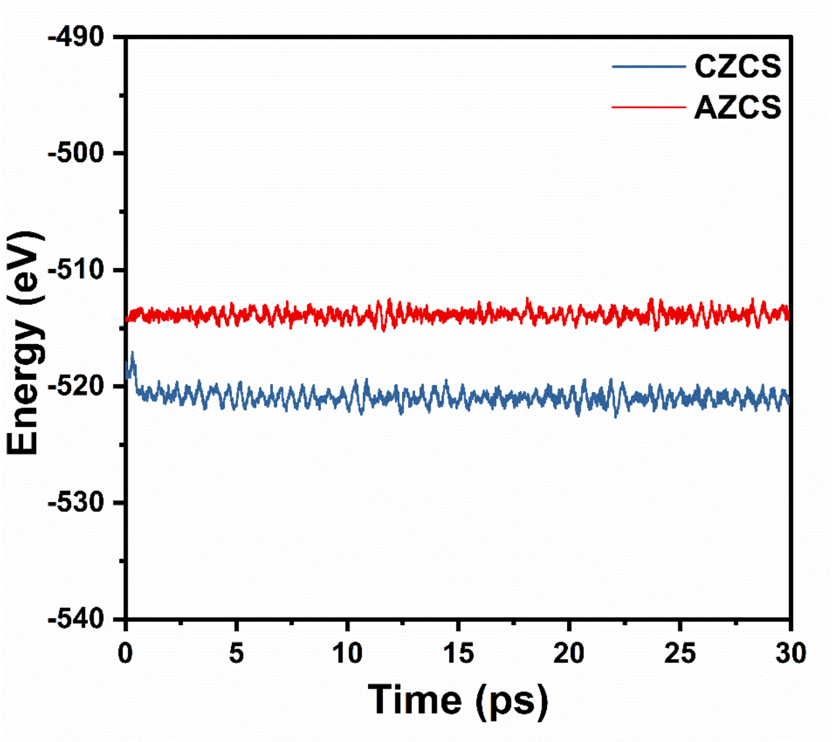

Fig. S2. DFT enery calculations. DFT energy of CZCS and AZCS as a function of time at 300 K.

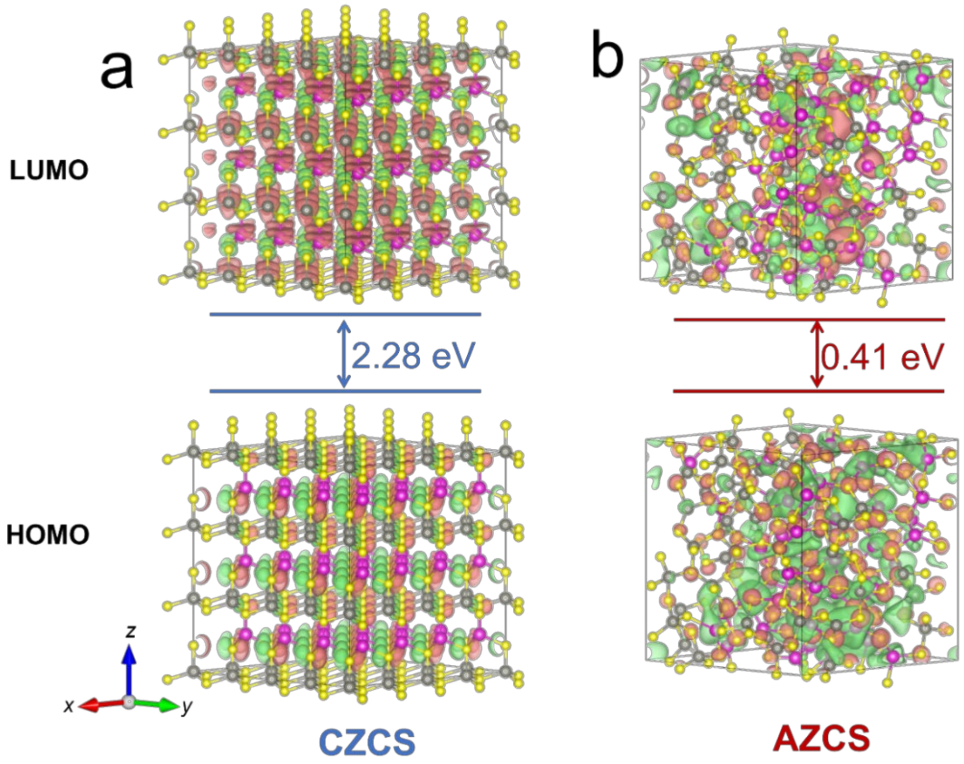

Fig. S3. HOMO and LUMO distribution calculations. **a** CZCS and **b** AZCS (Zn: grey spheres, Cd: pink spheres, S: yellow spheres).

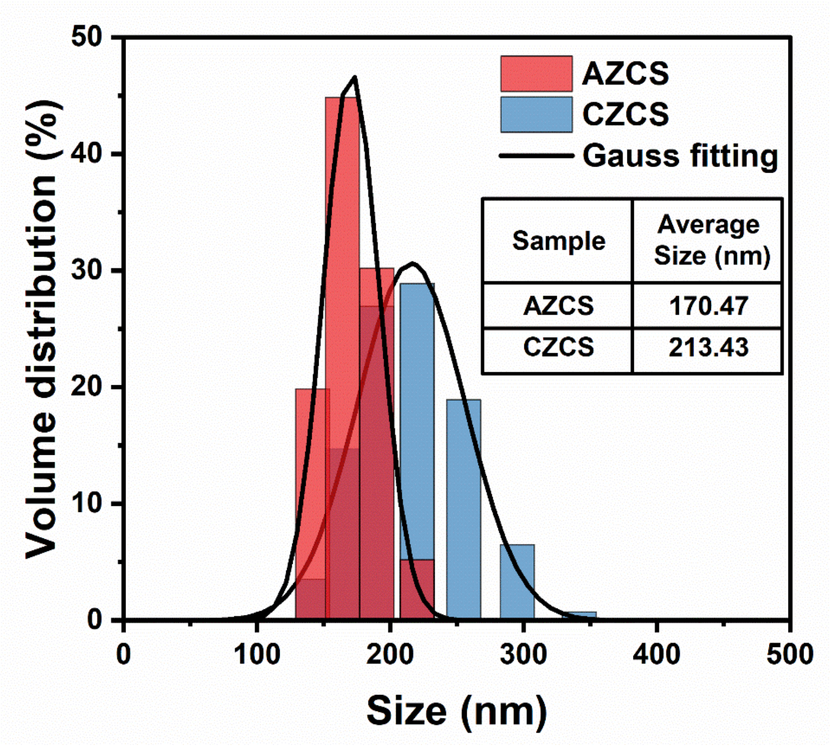

Fig. S4. Particle size distribution analysis. Volume distributions of AZCS and CZCS.

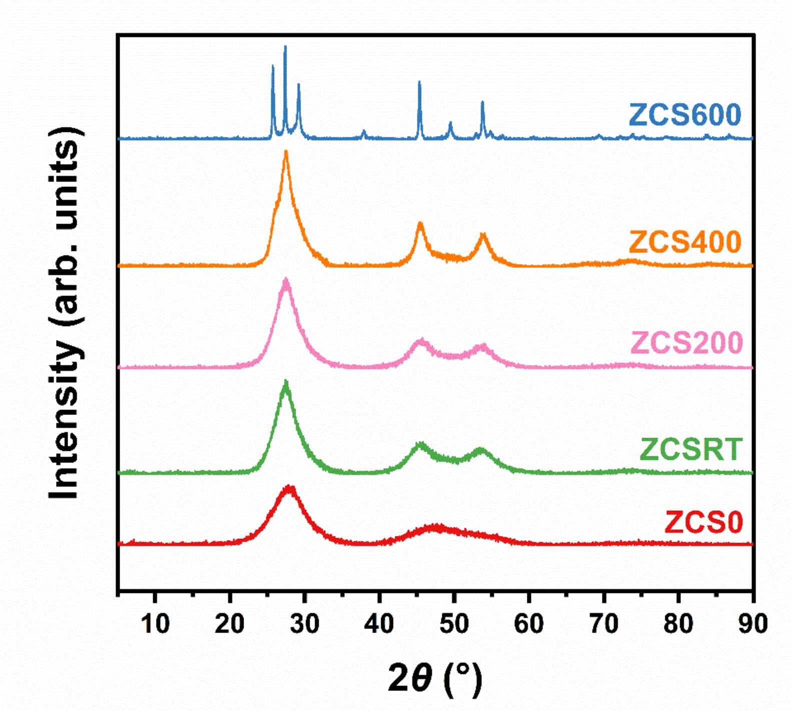

Fig. S5. XRD characterizations. XRD patterns of ZCS0, ZCSRT, ZCS200, ZCS400 and ZCS600.

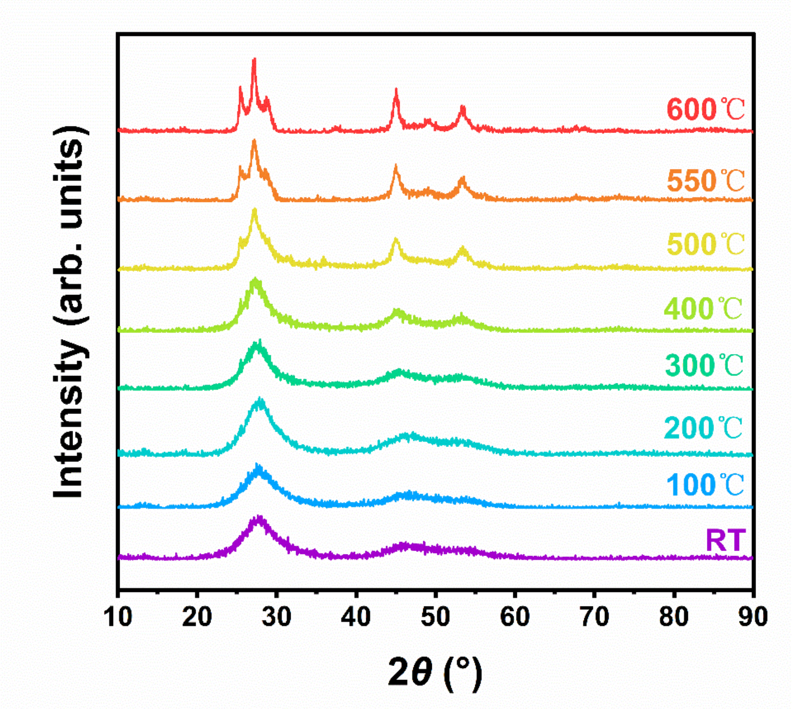

Fig. S6. In situ XRD characterizations. In situ XRD patterns of ZCS0 sintering from room temperature (RT) to 600 °C.

Supplementary Discussion

To unveil the physical and chemical changes of AZCS during sintering, thermogravimetric (TG) analysis with a heating rate of 2 °C/min was performed for AZCS. As demonstrated in Supplementary Fig. S7, it is obvious that a small amount of decomposition exists at 509 °C, and the mass retention is 88.85% at 600 °C, indicating that AZCS is relatively stable at this temperature. When the temperature is higher than 700 °C, serious decomposition of the material is observed (Supplementary Fig. S7).

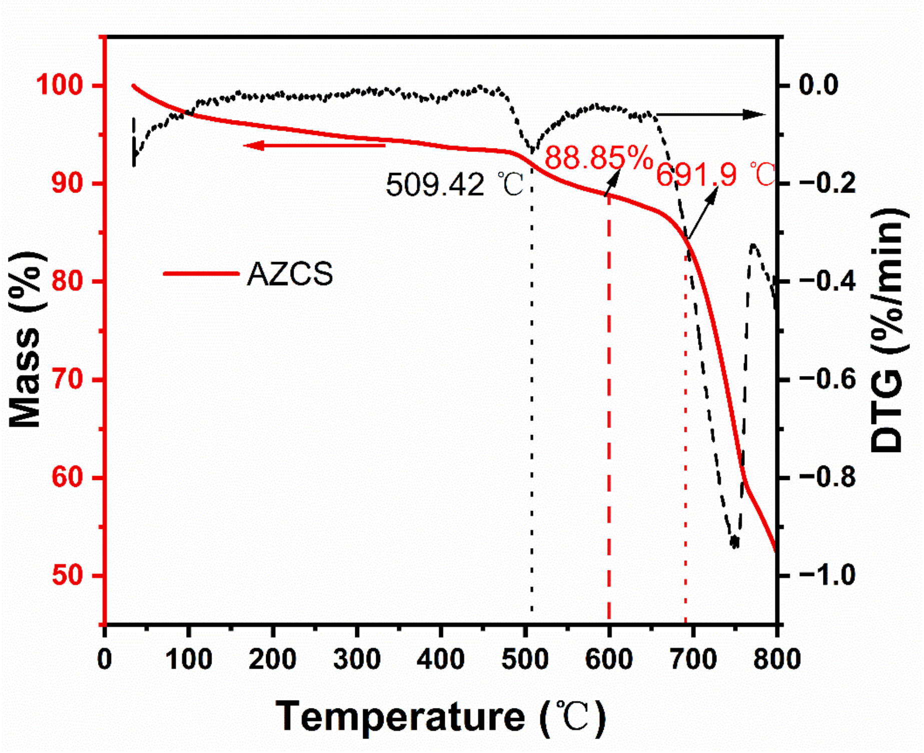

Fig. S7. TG analysis. TG curves of AZCS.

Supplementary Discussion

X-ray photoelectron spectroscopy (XPS) was conducted to characterize the surface compositions and chemical states of AZCS and CZCS. The XPS survey spectra (Supplementary Fig. S8a) of AZCS and CZCS demonstrate the presence of Zn, Cd and S elements. The O 1*s* peak is due to the surface adsorbed H_2O and CO_2 molecules, as evidenced by FTIR (Fig. 2q). The C 1*s* peak is the surface contaminated carbon for calibration. As shown in Supplementary Fig. S8b, the S $2p_{1/2}$ and S $2p_{3/2}$ peaks of CZCS are located at 163.04 and 161.86 eV, respectively, which are in accordance with the reported literature[1]. In comparison, the S $2p_{1/2}$ and S $2p_{3/2}$ peaks of AZCS are negatively shifted by about 0.09 and 0.13 eV, suggesting the generation of S-vacancies on the surface of AZCS[2]. It is worth noting that both AZCS and CZCS show two small S 2*p* peaks at lower binding energies, which should be assigned to unbounded S^{2-} or bonded to a single Zn/Cd (dissociative S^{2-})[3]. The absence of S 2*p* peaks at higher binding energy positions (>164.0 eV) proves that no S-O sulfone bridges are generated on the surface of both samples[2]. As observed in Supplementary Figs. S8c, d, the Zn 2*p* and Cd 3*d* peaks in AZCS also exhibit a slightly negative shift compared to its CZCS counterpart, which further supports the formation of S-vacancies on the surface of AZCS[4]. In addition to peaks for bond formation in ZnCdS, both Zn 2*p* and Cd 3*d* spectra exhibit peaks for dissociative ions[5], which is consistent with the S 2*p* spectra.

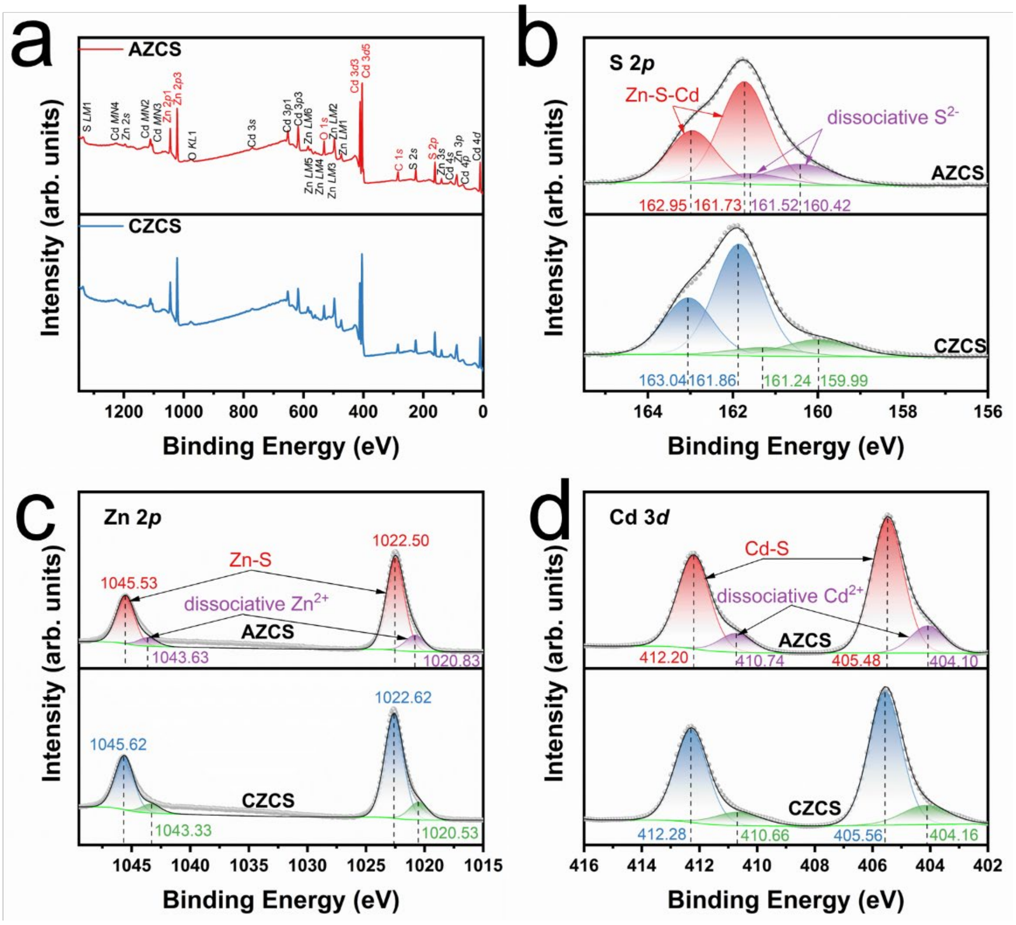

Fig. S8. XPS spectra. a XPS survey, **b** S 2*p*, **c** Zn 2*p* and **d** Cd 3*d* for AZCS and CZCS.

Supplementary Discussion

Since the interfacial binding between the photocatalyst and cocatalyst may also affect the photocatalytic activity, Raman spectroscopy was used to characterize the effect of Co-MoS_x on the vibration modes of AZCS and CZCS functional groups, respectively. As shown in Supplementary Fig. S9, although Co-MoS_x does not show its characteristic peaks on the Raman spectrum, it will affect the characteristic peaks of AZCS and CZCS, respectively. Since the intrinsic structures of AZCS and CZCS are different, the influence of Co-MoS_x on their Raman peaks is also different. To exclude the effect of cocatalyst on the enhanced photocatalytic activity of AZCS compared to CZCS, the influence trend of different cocatalysts on the photocatalytic H_2 evolution performance of AZCS and CZCS was also investigated. As shown in Supplementary Figs. S10, 11, when Co-MoS_x, MoS_2 and Pt were used as the cocatalysts, the ratios of the average H_2 evolution rates of AZCS and CZCS are 5.0, 4.7 and 4.03, respectively.

It is worth noting that without a cocatalyst, AZCS and CZCS also have photocatalytic H_2 evolution properties. In the first half an hour, the ratio of the H_2 evolution rates of AZCS and CZCS is 5.11, indicating that AZCS and CZCS have an inherent difference in photocatalytic H_2 evolution performance. Owing to the sluggish photocatalytic reaction without a cocatalyst, the difference between the photocatalytic performance of AZCS and CZCS gradually decreases. It should be mentioned that the photocatalytic H_2 production rate of AZCS gradually decreases with the irradiation time, while its CZCS counterpart exhibits a relatively stable photocatalytic H_2 production rate during the same irradiation time, indicating that AZCS without cocatalyst is less

stable than CZCS. These experimental results are consistent with the DFT calculations shown in Supplementary Fig. S2.

As shown in Supplementary Fig. S12, the color of AZCS without a cocatalyst appears dark green after 8 h of photocatalysis, while its counterpart with a Co-MoS_x as cocatalyst is still yellow, indicating that cocatalyst is essential to accelerate surface photocatalytic reactions and thus alleviating the destroy of the photocatalyst itself by the photogenerated charge carriers.

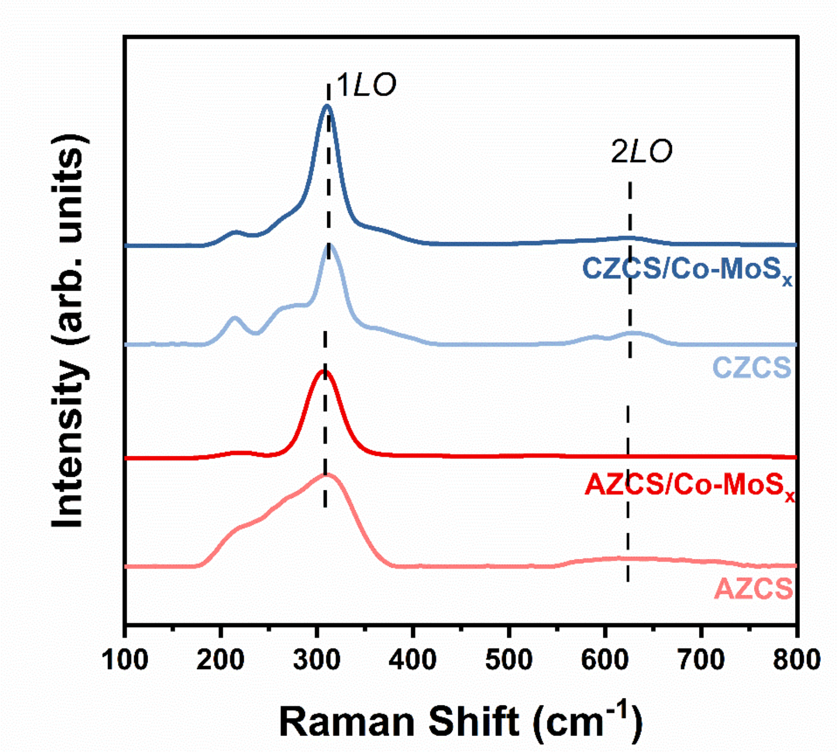

Fig. S9. Surface binding analysis. Raman spectra of AZCS, AZCS/Co-MoS_x, CZCS and CZCS/Co-MoS_x.

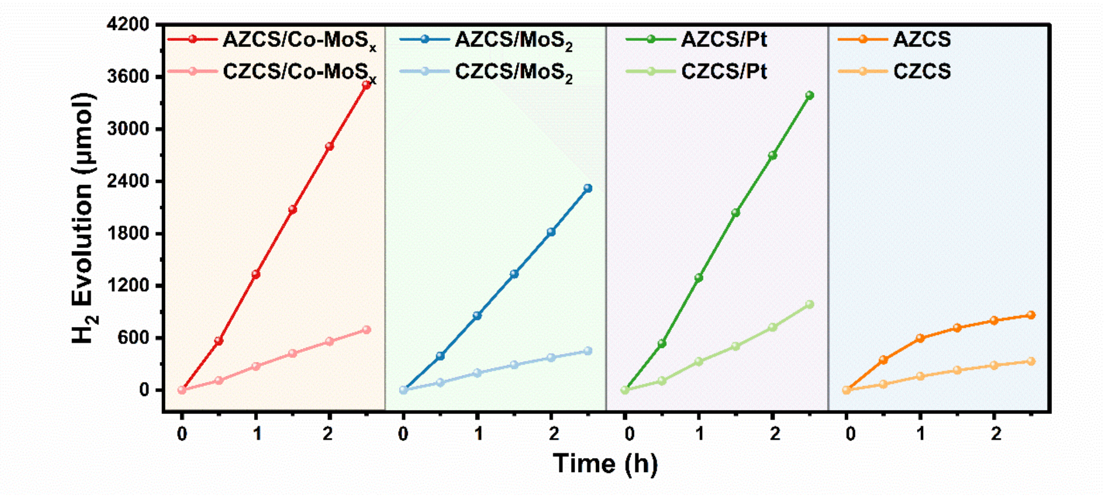

Fig. S10. Photocatalytic performance. Photocatalytic H_2 evolution of AZCS and CZCS loaded with different cocatalysts and without cocatalyst.

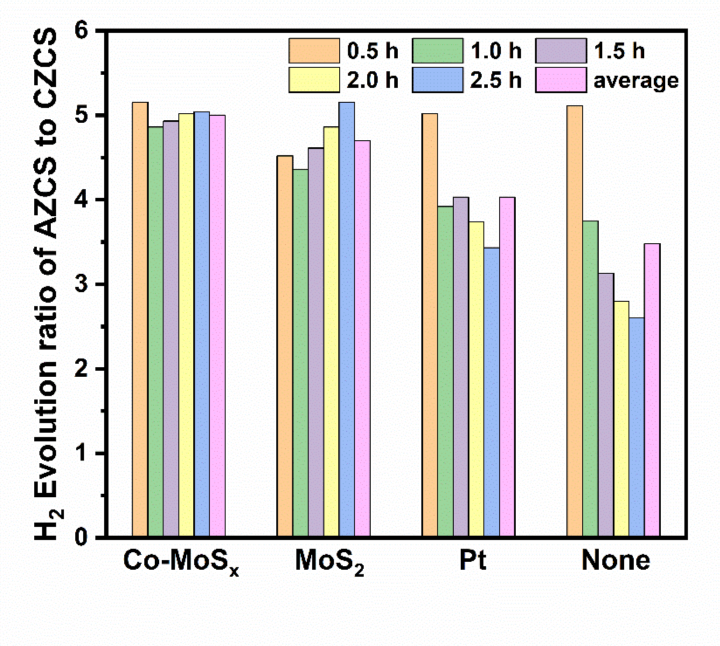

Fig. S11. Photocatalytic performance comparison with different cocatalysts. The ratio of H_2 evolution produced by AZCS and CZCS loaded with different cocatalysts at different time points.

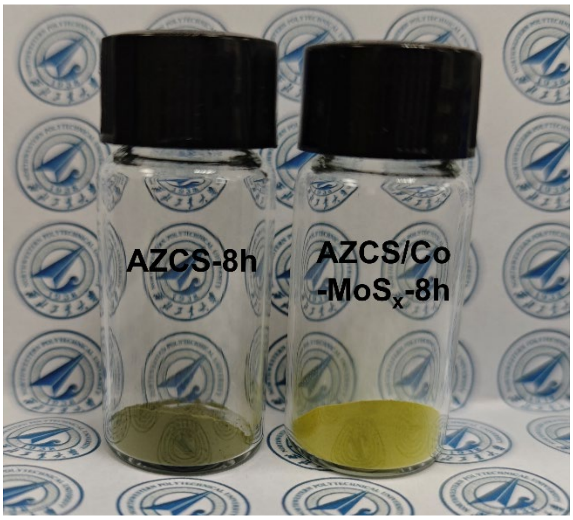

Fig. S12. Color comparison of different samples. Digital image shown the color of AZCS and AZCS/Co-MoS_x after 8 h of photocatalytic reaction.

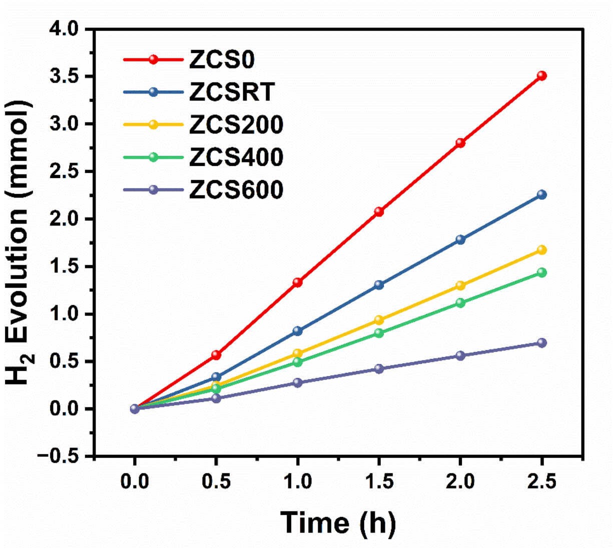

Fig. S13. Photocatalytic performance. H_2 generation within 2.5 hours of ZCS0, ZCSRT, ZCS200, ZCS400 and ZCS600.

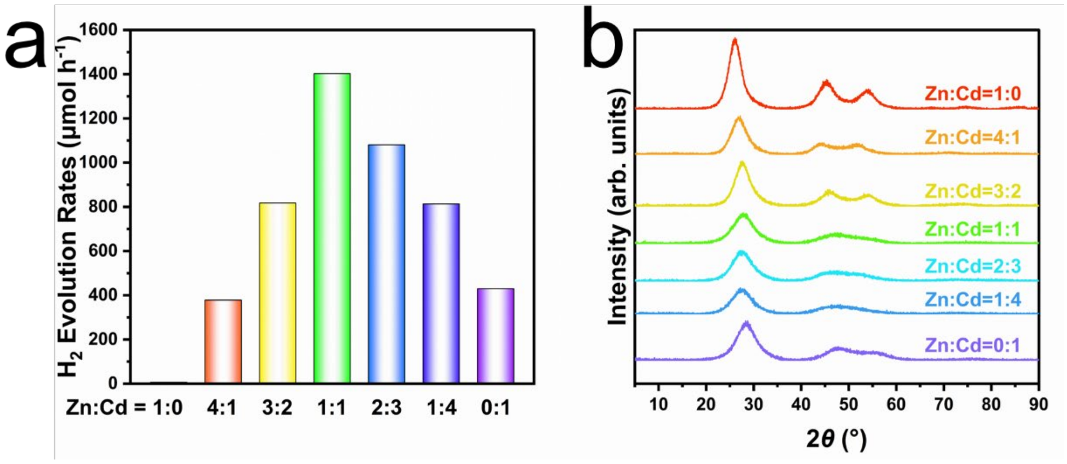

Fig. S14. Photocatalytic performances and crystal structures of Zn_xCd_yS (x:y = 1:0, 4:1, 3:2, 1:1, 2:3, 1:4, 0:1). a H_2 evolution rates and **b** XRD patterns.

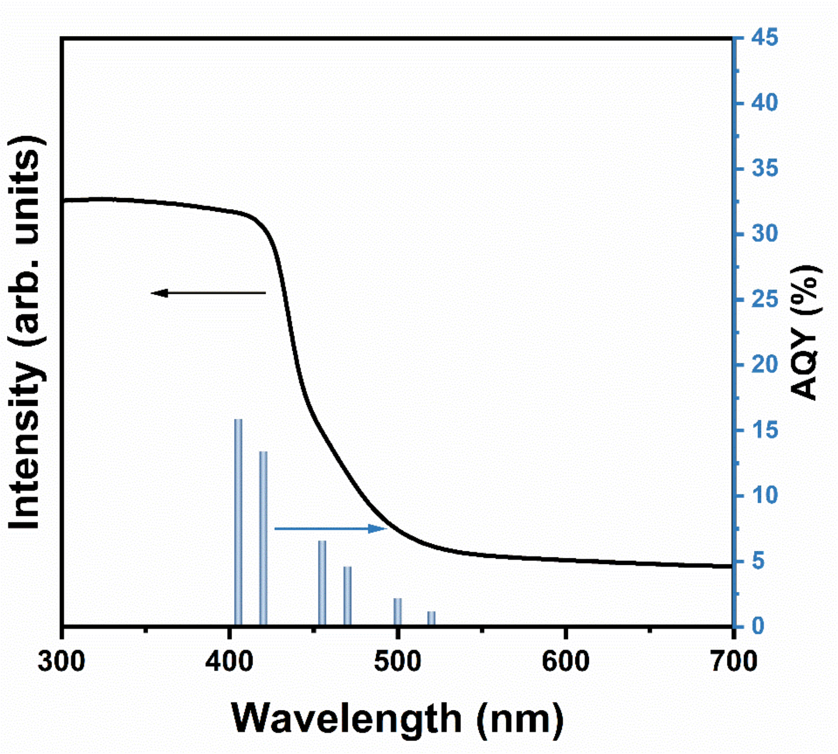

Fig. S15. Light absorption and AQY performance. Wavelength-dependent AQY of CZCS.

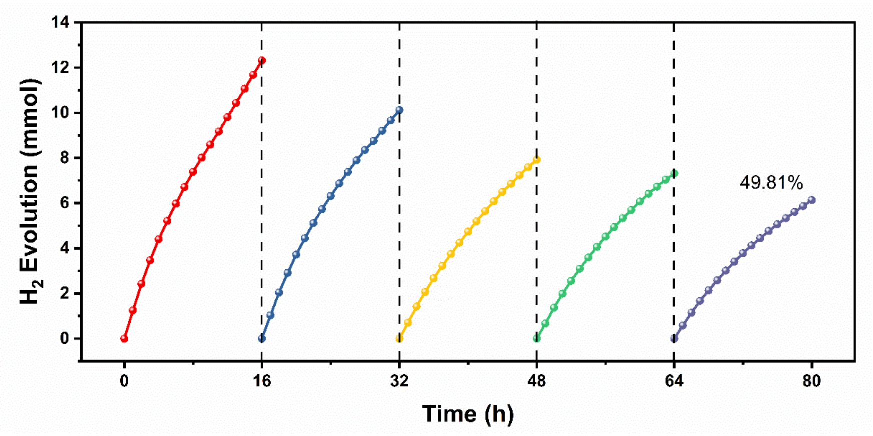

Fig. S16. Photocatalytic stability performance. Photocatalytic stability performance of Co-MoS_x/AZCS without adding cocatalyst per cycle.

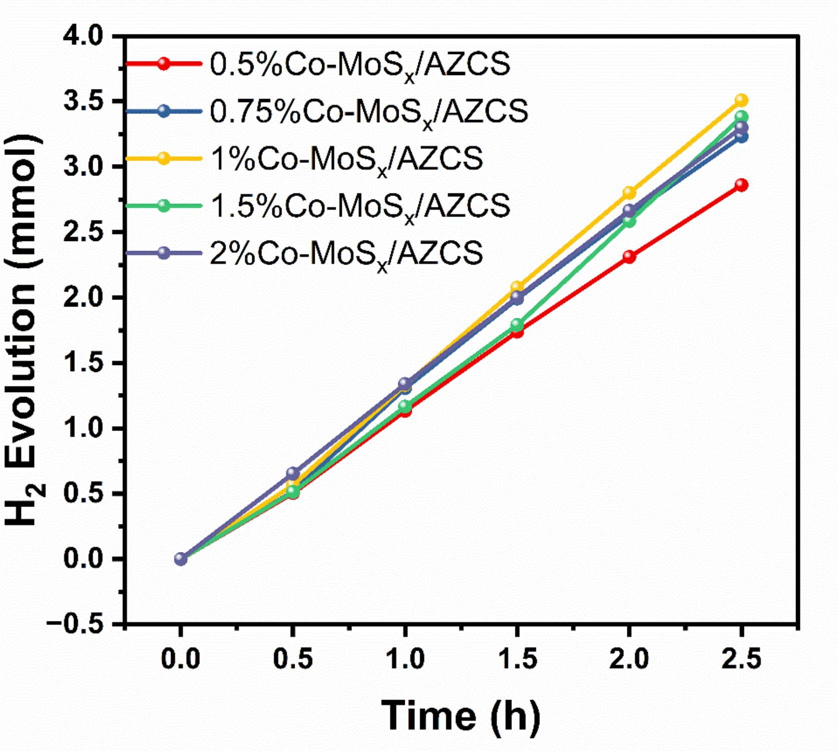

Fig. S17. Photocatalytic performance with different amounts of cocatalysts. Effect of different amounts of Co-MoS_x on the photocatalytic H_2 evolution performance of AZCS.

Supplementary Discussion

To further understand the underlying mechanism for the excellent photostability, SEM and XRD were used to characterize the changes in morphology and structure of AZCS without Co-MoS_x loading after 8 h of photocatalysis (denoted as AZCS-8h), AZCS photo-deposited with Co-MoS_x before photocatalysis measurement (denoted as AZCS/CMS), AZCS with Co-MoS_x added in each cycle during 5 cycles of photocatalytic reaction (denoted as AZCS/5-CMS), and AZCS with Co-MoS_x added only in the first cycle after 5 cycles of photocatalytic reaction (denoted as AZCS/CMS-5). As shown in the Figs. S18, 19, no obvious change can be observed in both SEM images and XRD patterns, indicating the excellent stability of our newly developed AZCS photocatalyst.

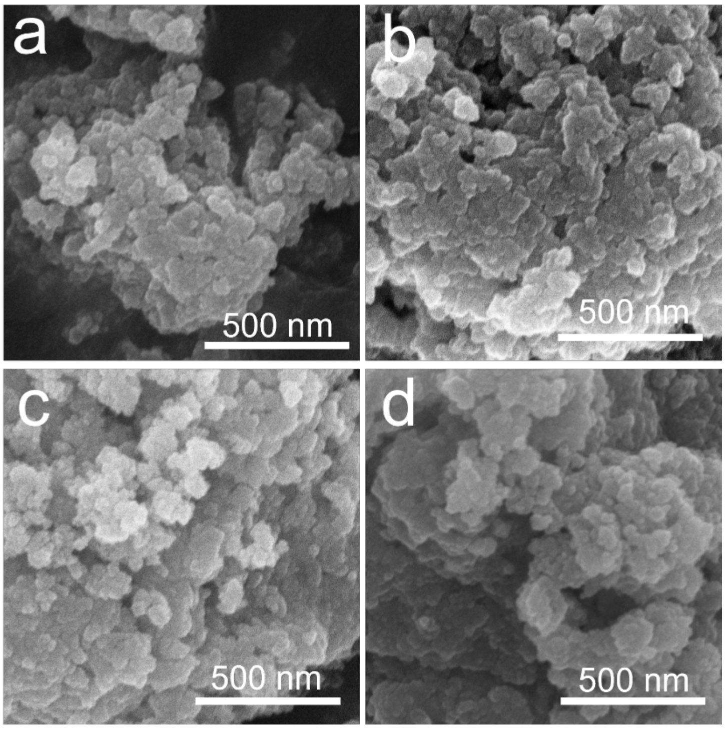

Fig. S18. SEM images of the photocatalysts. a AZCS-8h, **b** AZCS/CMS, **c** AZCS/5-CMS and **d** AZCS/CMS-5.

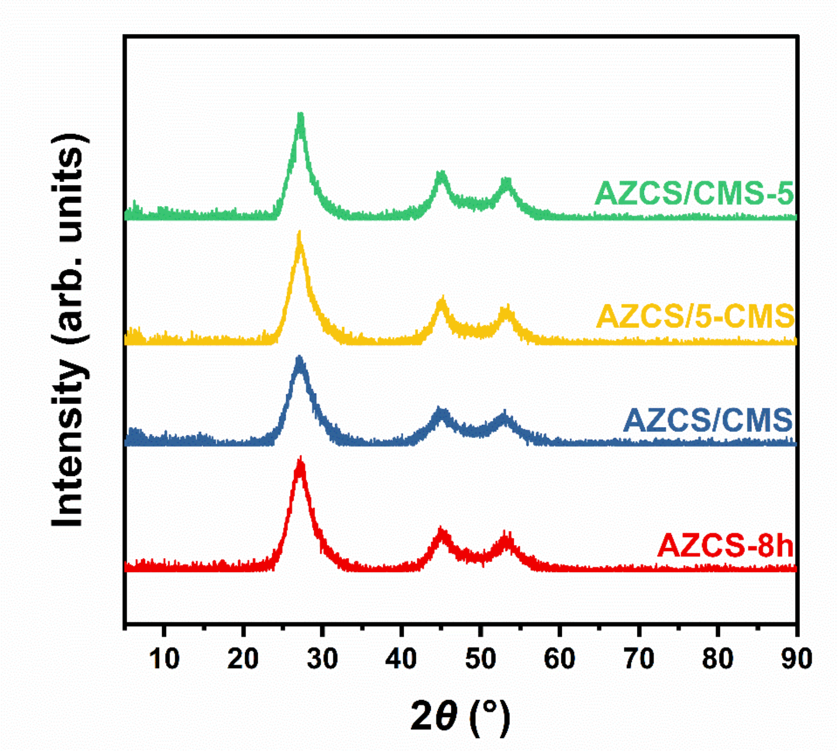

Fig. S19. XRD characterizations. XRD patterns of AZCS-8h, AZCS/CMS, AZCS/5-CMS and AZCS/CMS-5.

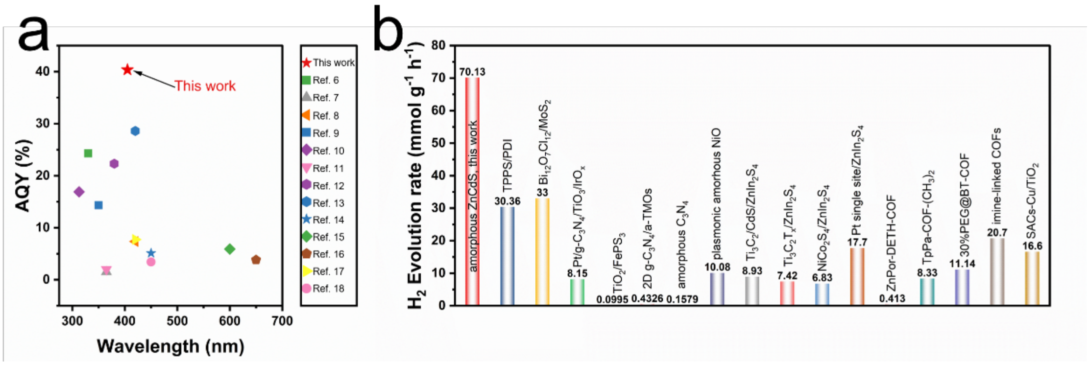

Fig. S20. Photocatalytic performance comparison of different potocatalysts. a AQY values[6-18], **b** H_2 evolution rate of the reported photocatalysts with built-in electric fields[6,16,19–21] or amorphous structures[22–24], as well as sulfide[2,18,25,26] and COF-based[27–30] photocatalysts.

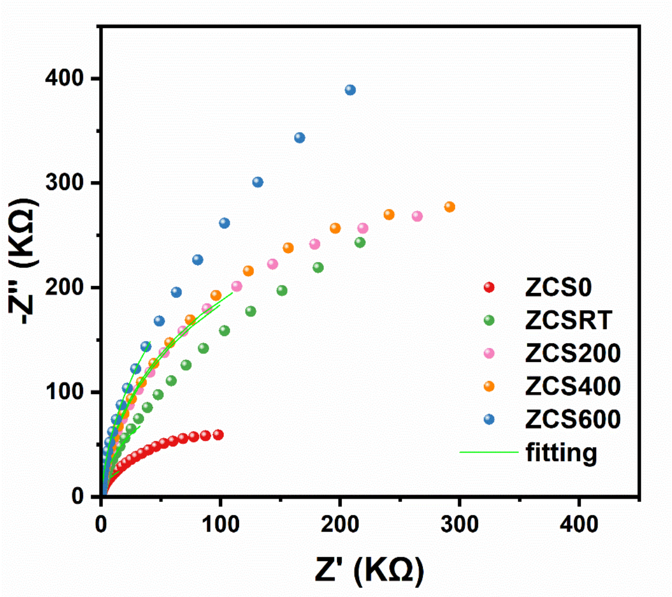

Fig. S21. EIS analysis. EIS plots of ZCS0, ZCSRT, ZCS200, ZCS400 and ZCS600.

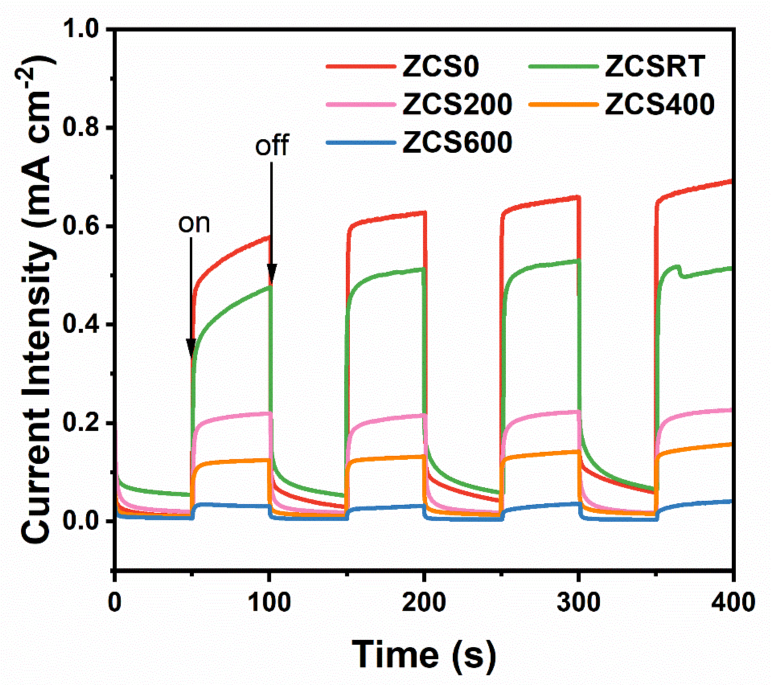

Fig. S22. Transient photocurrent responses in dark and under light. Transient photocurrent responses of ZCS0, ZCSRT, ZCS200, ZCS400 and ZCS600.

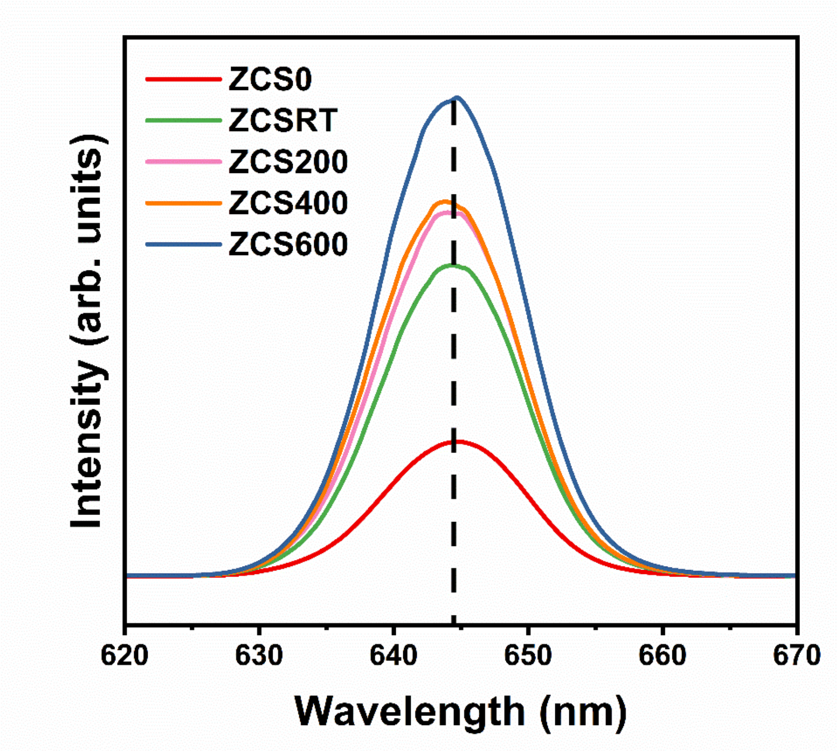

Fig. S23. PL analysis. PL spectra of ZCS0, ZCSRT, ZCS200, ZCS400 and ZCS600.

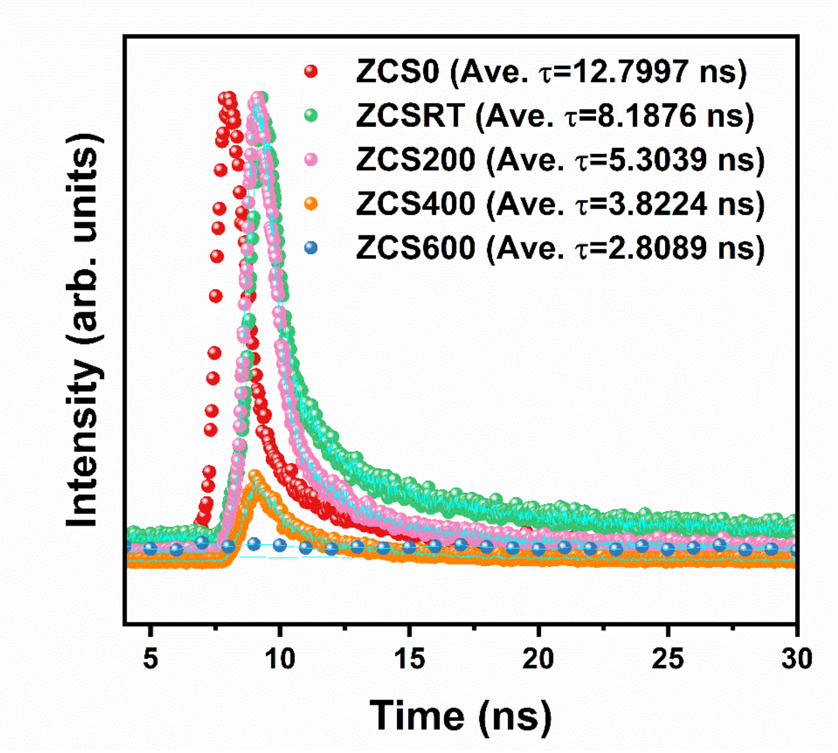

Fig. S24. PL lifetime analysis. PL lifetime of ZCS0, ZCSRT, ZCS200, ZCS400 and ZCS600.

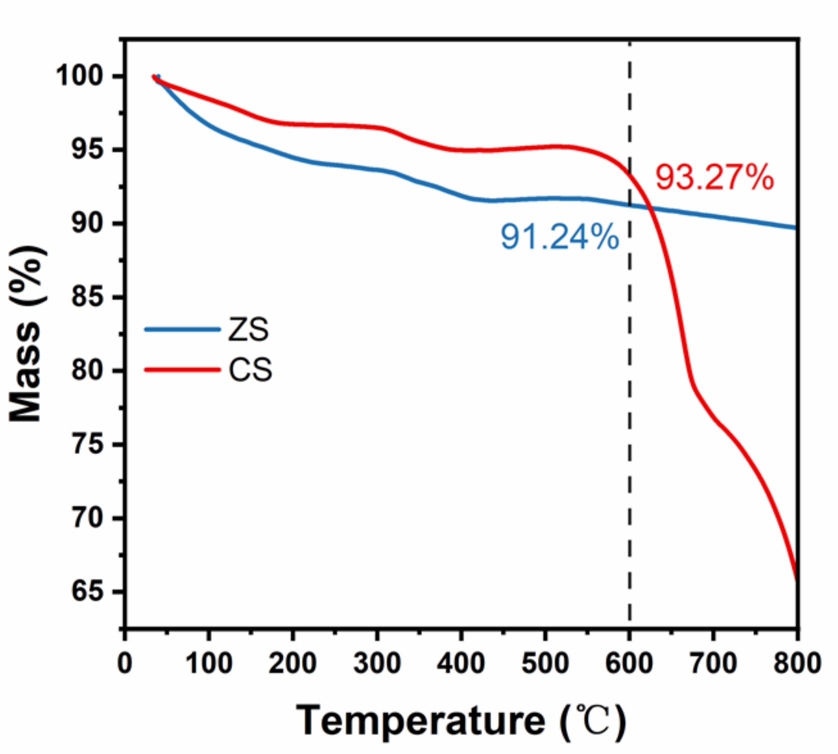

Fig. S25. TG analysis. TG curves of ZS and CS.

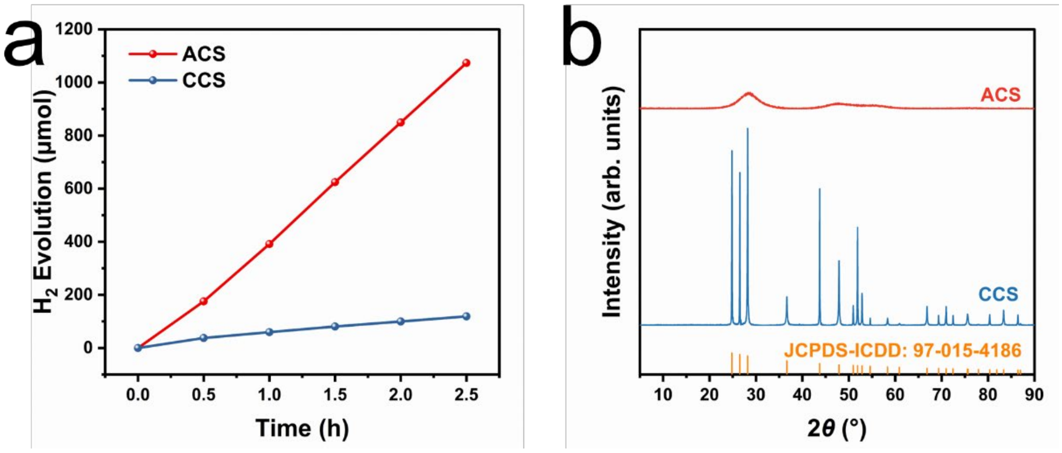

Fig. S26. Photocatalytic performances and crystal structures of ACS and CCS. a H_2 evolution generation within 2.5 hours and **b** XRD patterns.

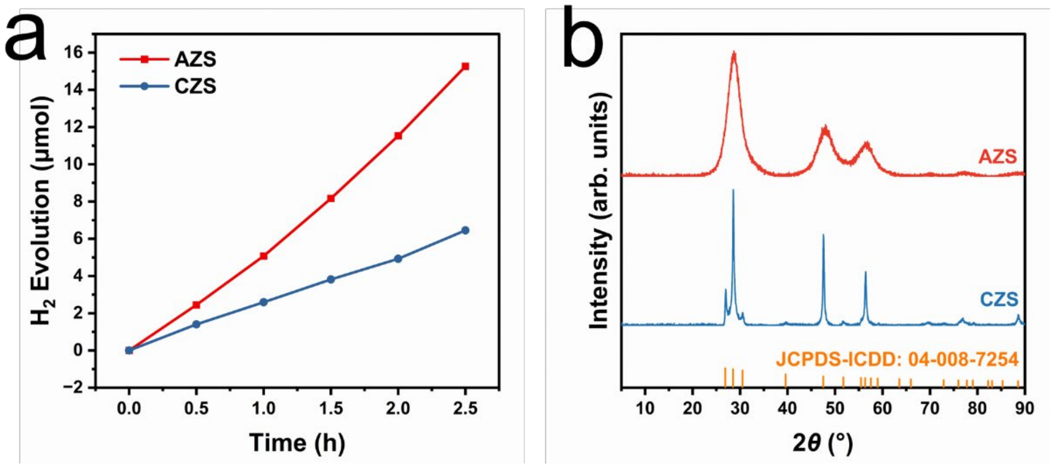

Fig. S27. Photocatalytic performances and crystal structures of AZS and CZS. a H_2 evolution generation within 2.5 hours and **b** XRD patterns.

Table S1. Summary of crystallinities of ZCS0, ZCSRT, ZCS200, ZCS400 and ZCS600.

**Sample**	ZCS0	ZCSRT	ZCS200	ZCS400	ZCS600
**Crystalline area**	100566	227895	317438	406843	584055
**Tatol area**	620775	702729	642978	619342	645222
**Crystallinity (W_c)**	16.20	32.43%	49.37%	65.68%	90.52%
**Error**	1.62%	1.39%	2.26%	2.90%	1.25%

Table S2. The calculated AQY of AZCS and CZCS at different wavelengths.

	AZCS		CZCS	
**Wavelength**	H_2 evolution in 30 min (μmol)	Light power (mW cm^{-2})	H_2 evolution in 30 min (μmol)	Light power (mW cm^{-2})
**405 nm**	108.88	4.52	106.22	11.19
**420 nm**	159.87	6.70	177.64	21.46
**455 nm**	145.06	9.29	106.17	24.13
**470 nm**	101.38	9.14	66.34	20.77
**500 nm**	38.79	6.88	19.76	12.13
**520 nm**	22.79	5.91	10.27	11.16

$$\begin{aligned}\mathrm{AQY} &= \frac{\text{the number of reacted electrons}}{\text{the number of incident photons}}\times 100\% \\ &= \frac{2\times\text{the number of }\mathrm{H_2}\text{ molecules}}{\text{the number of incident photons}}\times 100\% \\ &= \frac{2\times M\times N_{\mathrm{A}}\times H\times c}{S\times P\times t\times\lambda}\times 100\%\end{aligned}$$

Such as, when λ = 405 nm:

$$\begin{aligned}\mathrm{AQY(AZCS)} &= \frac{2\times 108.88\times 10^{-6}\times 6.02\times 10^{23}\times 6.626\times 10^{-34}\times 3\times 10^{8}}{0.00452\times 19.6\times 1800\times 405\times 10^{-9}}\times 100\% \\ &= 40.35\%\end{aligned}$$

$$\begin{aligned}\mathrm{AQY(CZCS)} &= \frac{2\times 106.22\times 10^{-6}\times 6.02\times 10^{23}\times 6.626\times 10^{-34}\times 3\times 10^{8}}{0.01119\times 19.6\times 1800\times 405\times 10^{-9}}\times 100\% \\ &= 15.90\%\end{aligned}$$

Supplementary Discussion

To further understand the change of Co-MoS_x cocatalyst on AZCS with and without additional Co-MoS_x cocatalyst in each cycle during photocatalytic H_2 production, ICP was used to investigate the amount of cocatalyst in AZCS/Co-MoS_x and the supernatant liquid after each hydrogen evolution cycle. As listed in Supplementary Table S3, with additional Co-MoS_x cocatalyst in each cycle, the Mo content in AZCS/Co-MoS_x increases after 1, 5, and 10 cycles, and the supernatant liquid after 1 and 10 cycles also contains a small amount of Mo. However, without additional Co-MoS_x cocatalyst in each cycle, almost no Mo signals can be detected in the supernatant liquid after photocatalytic hydrogen evolution for 5 cycles. In addition, the Cd contents in the photocatalyst and the supernatant liquid are almost unchanged from the 1st to the 10th cycle, indicating that AZCS itself is stable during the photocatalytic process. The above results indicate that not all Co-MoS_x are attached to AZCS during the photo-deposition process, and the loading amount of Co-MoS_x on AZCS is relatively stable during photocatalytic hydrogen evolution. However, the Co-MoS_x cocatalyst gradually lose activity for H_2 evolution (Supplementary Fig. S16). During the photocatalytic process, the reducible support easily forms overlayers on the surface of the cocatalyst, thereby affecting the redox reaction on the surface of the photocatalyst[31,32]. Therefore, adding an appropriate amount of Co-MoS_x cocatalyst during the photocatalytic process is necessary to maintain the activity for hydrogen evolution, thereby achieving excellent long-term stability.

Table S3. Summary of Cd and Mo contents in the supernatant liquid and photocatalyst after photocatalytic cycle.

**Sample**	Photocatalyst			Supernatant liquid		
**cycle**	1	5	10	1	10	5/N
**Cd (mg)**	20.23	19.64	20.25	0.108	0.0615	0.0716
**Mo (μg)**	36	48	84	13.6	8	1

5/N represents the supernatant liquid after the fifth hydrogen evolution cycle of the sample without adding Co-MoS_x during each cycle.

Table S4. Summary of the R_{ct} values of ZCS0, ZCSRT, ZCS200, ZCS400 and ZCS600.

**Sample**	ZCS0	ZCSRT	ZCS200	ZCS400	ZCS600
$\boldsymbol{R_{ct}}$ **(KΩ)**	62.282	204.25	506.93	516.95	770.12

Supplementary References

1. Zeng, R. *et al.* Versatile Synthesis of Hollow Metal Sulfides via Reverse Cation Exchange Reactions for Photocatalytic CO_2 Reduction. *Angew. Chem. Int. Ed.* **60**, 25055–25062 (2021).
2. Shi, X. *et al.* Protruding Pt single-sites on hexagonal $ZnIn_2S_4$ to accelerate photocatalytic hydrogen evolution. *Nat. Commun.* **13**, 1287 (2022).
3. Liang, X. *et al.* A highly efficient polysulfide mediator for lithium-sulfur batteries. *Nat. Commun.* **6**, 5682 (2015).
4. Zhou, H. *et al.* A General Polymer-Oriented Acid-Mediated Self-Assembly Approach toward Crystalline Mesoporous Metal Sulfides. *Small* **17**, 2100428 (2021).
5. Wu, C. *et al.* A New Scalable Preparation of Metal Nanosheets: Potential Applications for Aqueous Zn-Ion Batteries Anode. *Adv. Funct. Mater.* **30**, 2003187 (2020).
6. Moon, H. S. *et al.* Spatial Separation of Cocatalysts on Z-Scheme Organic/Inorganic Heterostructure Hollow Spheres for Enhanced Photocatalytic H_2 Evolution and In-Depth Analysis of the Charge-Transfer Mechanism. *Adv. Mater.* **35**, 2200172 (2023).
7. Liu, Y. *et al.* Atomically Dispersed Silver-Cobalt Dual-Metal Sites Synergistically Promoting Photocatalytic Hydrogen Evolution. *Adv. Funct. Mater.* **33**, 2301840 (2023).

8. Huang, K. *et al.* Boosting Photocatalytic Hydrogen Evolution Through Local Charge Polarization in Chemically Bonded Single-Molecule Junctions Between Ketone Molecules and Covalent Organic Frameworks. *Adv. Funct. Mater.* **33**, 2307300 (2023).

9. Liu, Y. *et al.* Regulating electron−hole separation to promote photocatalytic H_2 evolution activity of nanoconfined Ru/Mxene/TiO_2 catalysts. *ACS Nano* **14**, 14181–14189 (2020).

10. Dai, B. *et al.* Construction of Infrared-Light-Responsive Photoinduced Carriers Driver for Enhanced Photocatalytic Hydrogen Evolution. *Adv. Mater.* **32**, 1906361 (2020).

11. Ayala, P. *et al.* The Emergence of 2D Building Units in Metal-Organic Frameworks for Photocatalytic Hydrogen Evolution: A Case Study with COK-47. *Adv. Energy Mater.* **13**, 2300961 (2023).

12. Jia, G. *et al.* Wet-chemistry hydrogen doped TiO_2 with switchable defects control for photocatalytic hydrogen evolution. *Matter* **5**, 206–218 (2022).

13. Zhou, Q., Guo, Y. & Zhu, Y. Photocatalytic sacrificial H_2 evolution dominated by micropore-confined exciton transfer in hydrogen-bonded organic frameworks. *Nat. Catal.* **6**, 574–584 (2023).

14. Ma, S. *et al.* Photocatalytic Hydrogen Production on a sp^2 -Carbon-Linked Covalent Organic Framework. *Angew. Chem. Int. Ed.* **134**, e202208919 (2022).

15. Yang, Y. *et al.* Enhanced Photocatalytic Hydrogen Evolution from Organic Ternary Heterojunction Nanoparticles Featuring a Compact Alloy-Like Phase. *Adv. Funct. Mater.* **33**, 2209643 (2023).

16. Yang, J., Jing, J., Li, W. & Zhu, Y. Electron Donor–Acceptor Interface of TPPS/PDI Boosting Charge Transfer for Efficient Photocatalytic Hydrogen Evolution. *Adv. Sci.* **9**, 2201134 (2022).

17. Wang, W. *et al.* Near-Field Drives Long-Lived Shallow Trapping of Polymeric C_3N_4 for Efficient Photocatalytic Hydrogen Evolution. *Adv. Funct. Mater.* **31**, 2103978 (2021).

18. Bai, J. *et al.* Regulating interfacial morphology and charge-carrier utilization of Ti_3C_2 modified all-sulfide CdS/$ZnIn_2S_4$ S-scheme heterojunctions for effective photocatalytic H_2 evolution. *J. Mater. Sci. Technol.* **112**, 85–95 (2022).

19. Li, J., Zhan, G., Yu, Y. & Zhang, L. Superior visible light hydrogen evolution of Janus bilayer junctions via atomic-level charge flow steering. *Nat. Commun.* **7**, 11480 (2016).

20. Xia, B. *et al.* TiO_2/$FePS_3$ S-Scheme Heterojunction for Greatly Raised Photocatalytic Hydrogen Evolution. *Adv. Energy Mater.* **12**, 2201449 (2022).

21. Lee, B. H. *et al.* Reversible and cooperative photoactivation of single-atom Cu/TiO_2 photocatalysts. *Nat. Mater.* **18**, 620–626 (2019).

22. Zhou, B. X. *et al.* Generalized Synthetic Strategy for Amorphous Transition Metal Oxides-Based 2D Heterojunctions with Superb Photocatalytic Hydrogen and Oxygen Evolution. *Adv. Funct. Mater.* **31**, 2009230 (2021).

23. Kang, Y. *et al.* An Amorphous Carbon Nitride Photocatalyst with Greatly Extended Visible-Light-Responsive Range for Photocatalytic Hydrogen Generation. *Adv. Mater.* **27**, 4572–4577 (2015).

24. Lin, Z., Du, C., Yan, B., Wang, C. & Yang, G. Two-dimensional amorphous NiO as a plasmonic photocatalyst for solar H_2 evolution. *Nat. Commun.* **9**, 4036 (2018).

25. Guo, Y. *et al.* Concentrating electron and activating H-OH bond of absorbed water on metallic $NiCo_2S_4$ boosting photocatalytic hydrogen evolution. *Nano Energy* **95**, 107028 (2022).

26. Su, T. *et al.* Sulfur Vacancy and $Ti_3C_2T_x$ Cocatalyst Synergistically Boosting Interfacial Charge Transfer in 2D/2D $Ti_3C_2T_x/ZnIn_2S_4$ Heterostructure for Enhanced Photocatalytic Hydrogen Evolution. *Adv. Sci.* **9**, 2103715 (2022).

27. Chen, R. *et al.* Rational design of isostructural 2D porphyrin-based covalent organic frameworks for tunable photocatalytic hydrogen evolution. *Nat. Commun.* **12**, 1354 (2021).

28. Sheng, J. L. *et al.* Effect of Different Functional Groups on Photocatalytic Hydrogen Evolution in Covalent-Organic Frameworks. *ChemCatChem* **11**, 2313–2319 (2019).

29. Zhou, T. *et al.* PEG-stabilized coaxial stacking of two-dimensional covalent organic frameworks for enhanced photocatalytic hydrogen evolution. *Nat. Commun.* **12**, 3934 (2021).

30. Yang, J. *et al.* Protonated Imine-Linked Covalent Organic Frameworks for Photocatalytic Hydrogen Evolution. *Angew. Chem. Int. Ed.* **60**, 19797–19803 (2021).

31. Monai, M. *et al.* Restructuring of titanium oxide overlayers over nickel nanoparticles during catalysis. *Science* **380**, 644–651 (2023).

32. Frey, H., Beck, A., Huang, X., van Bokhoven, J. A. & Willinger, M. G. Dynamic interplay between metal nanoparticles and oxide support under redox conditions. *Science.* **376**, 982–987 (2022).
