# Supplementary material for: Induced dipole moments in amorphous ZnCdS catalysts facilitate photocatalytic H2 evolution
Source: Nat Commun. 2024 Mar 23;15:2600. doi: 10.1038/s41467-024-47022-z (PMC10960824; doi:10.1038/s41467-024-47022-z)
Supplement: Supplementary file 3 — Description of Additional Supplementary Files [file 41467_2024_47022_MOESM3_ESM.pdf]

Description of Additional Supplementary Files

File Name: Supplementary Movie 1
Description: hydrogen evolution of Co-MoSx/AZCS under Xe lamp light.

File Name: Supplementary Movie 2
Description: hydrogen evolution of Co-MoSx/AZCS under natural sunlight.

File Name: Supplementary Movie 3
Description: a flexible Co-MoSx/AZCS film with repeated bending.

File Name: Supplementary Movie 4
Description: hydrogen evolution of a flexible Co-MoSx/AZCS film under Xe lamp light.

File Name: Supplementary Movie 5
Description: hydrogen evolution of a flexible Co-MoSx/AZCS film under natural sunlight.
